# Diabetes Treated by Alkalies

**Published:** 1844-10-19

**Authors:** 


### DIABETES TREATED BY ALKALIES.

MM. Miale and Contour narrated a case of diabetes mellitus cured by the use of alkalies and sudorifics. The patient, a man aged forty-three, had been labouring under diabetes for eighteen months, and was in the following state:—Extreme prostration and emaciation, great weakness, appetite good, digestion easy, thirst intense, dryness of the mouth, although he drank five or six quarts a day. His urine was abundant, and the quantity was always in relation to the fluid he introduced into the economy. It was acid and nearly colourless; density 1,035; it contained a little more than nine drachms of sugar for each quart. After giving, without any result, the chloride of sodium during fifteen days, the internal administration of alkalies was commenced, as also the use of flannel, of vapour-baths, and of highly-animalised diet. One drachm of bicarbonate of soda and eighteen grains of calcined magnesia were given daily, during eight days. The dose of bicarbonate was then progressively raised to one drachm and a half, to two and a half, and, lastly, to three. The doses of the magnesia remained the same. This treatment lasted a month, and was followed by complete success. The quantity of sugar contained in the urine gradually decreased, and when the fluids of the economy had recovered their alkaline properties, it entirely disappeared. At the time the patient was cured, and eating every day a pound of bread along with a pint of milk. He still, however, continued the use of alkalies, and it was impossible to say whether the symptoms might not return, were their administration suspended.—*Ibid.*

